# Early Prediction and Streamline of Nucleophosmin Mutation Status in Acute Myeloid Leukemia Using Cup-Like Nuclear Morphology

**DOI:** 10.3390/medicina60091443

**Published:** 2024-09-04

**Authors:** Ljubomir Jakovic, Vesna Djordjevic, Nada Kraguljac Kurtovic, Marijana Virijevic, Mirjana Mitrovic, Lazar Trajkovic, Ana Vidovic, Andrija Bogdanovic

**Affiliations:** 1 Clinic of Hematology, University Clinical Center of Serbia, Koste Todorovica 2, 11000 Belgrade, Serbia; djvesna.kcs@gmail.com (V.D.); nada_kraguljac@yahoo.com (N.K.K.); marijana.virijevic@yahoo.com (M.V.); mirjanamitrovic777@gmail.com (M.M.); trajkovic.lazar33@gmail.com (L.T.); vidana103@gmail.com (A.V.); ebogdano@eunet.rs (A.B.); 2 Medical Faculty, University of Belgrade, Dr Subotica 8, 11000 Belgrade, Serbia

**Keywords:** cup-like blast, acute myeloid leukemia, *NPM1* mutation

## Abstract

*Background and Objectives*: With the advent of novel therapies for nucleophosmin gene (*NPM1*)-mutated acute myeloid leukemia (AML), there is a growing need for the reliable prediction of *NPM1* mutations. This study explored the role of cytomorphological features in the early prediction of *NPM1*-mutated AML. *Materials and Methods*: Altogether, 212 de novo AML cases with normal karyotypes, diagnosed and treated at a single institution within 5 years (2018–2023), were retrospectively evaluated. A final diagnosis of *NPM1*-mutated AML, based on the World Health Organization (WHO) integrated criteria, including real-time based identification of *NPM1* mutation and normal karyotype, was established in 83/212 (39.15%) cases. *Results*: Cup-like blasts (CLBs), a cytomorphological feature suggestive of *NPM1*-mutated AML, were detected in 56/83 (67%) patients. Most cases (44/56, 78.6%) had CLB ≥ 10%. In total, 27 of 83 AML *NPM1*-mutated patients had no CLB morphology (missed call). Additionally, two of 212 had CLB morphology without confirmed *NPM1* mutation (wrong call). The positive/negative predictive values of cytomorphological evaluation for CLB ≥ 10% were 95.7%/75.6%, with sensitivity/specificity of 53%/98.5%, while the accuracy was 80.7%. We noted an increased percentage of CLBs (≥15%) in 77.8% and 50% of patients with AML without and with granulocytic maturation, respectively (the specificity for *NPM1* mutation prediction was 100%). CLB was associated with fms-like tyrosine kinase 3 (*FLT3*) mutation (*p* = 0.03), but, without statistical significance for CLB ≥ 10% and CLB ≥ 15%. *Conclusions*: Our investigation confirmed that the morphological identification of CLB at diagnosis represents a reliable and easily reproducible tool for the early prediction of *NPM1* mutations, enabling a streamlined genetic work-up for its confirmation. This may facilitate considering the early administration of individualized therapies by clinicians for specific patients.

## 1. Introduction

Current classification proposals for acute myeloid leukemia (AML) are based on the complete integration of all diagnostic methods and depend on recent technological advances. Nevertheless, the morphological identification of cellular pathology remains a fundamental starting point in stepwise diagnostics. Further investigations through immunophenotyping, cytogenetics, and molecular genetics will provide a better understanding of leukemia biology, and the findings of these investigations will enable further prognostic stratification [1,2]. 

Both the International Consensus Classification (ICC) and World Health Organization (WHO) 5th classification postulate that abnormal morphology is a result of disturbed cellular biology, driven by somatic mutations or the expression of altered genes, thereby focusing more on extensive genetic profiling to describe disease entities. These genetic events could be used as specific biomarkers in the diagnostic process, and as a basis for personalized treatment and molecular-sensitive monitoring of diseases [1,2,3]. 

Mutations in the nucleophosmin (*NPM1*) gene are the most common genetic aberrations in patients with AML with normal karyotype [4]. In both the classifications, they are considered “recurrent cytogenetic aberrations” (RCA, WHO 5th) or “defining genetic abnormalities” (DCA, ICC), where a genetic lesion itself defines the disease [1,2,5].

Previously, the 2016 revision of the WHO classification required the presence of ≥20% cup-like blasts (CLBs) as the diagnostic criterion. However, the ICC has determined a blast count of at least 10% for *NPM1*-mutated AML, while the WHO 5th classification abolished it and instead considered morphological and genetic correlations [1,2,6]. 

*NPM1*-mutated AML is a specific leukemia type characterized by the disturbance of nuclear–cytoplasmic transport [7]. Since it was first described, this mutation has frequently been associated with a distinct morphological appearance of prominent nuclear membrane invaginations resembling cup-like nuclear pockets occupied by the reticulum, mitochondria, and occasional lysosomes [8,9]. 

The *NPM1* mutation often harbors an fms-like tyrosine kinase 3-internal tandem duplication (*FLT3*-ITD) mutation and is associated with a higher white blood cell (WBC) count, prominent blast fraction, and the absence of CD34 and/or human leukocyte antigen (HLA)-DR expression, occurring more frequently in females [4,10,11,12]. 

According to the current European LeukemiaNet (ELN) AML proposal, *NPM1* mutations in the absence of *FLT3*-ITD are a favorable finding [5]. It enables the use of more sensitive genetic testing for minimal residual disease (MRD) compared to classical leukemia-associated immunophenotype MRD detection. Contemporary real-time polymerase chain reaction (PCR) tests can improve MRD sensitivity to a level at least one log deeper than conventional flow cytometry-based assays [13].

Moreover, the earlier detection of *NPM1*-mutated AML has become more important as venetoclax-based therapies, new menin, and exportin 1 (XPO1) inhibitors can disrupt genetically rearranged molecular perturbations within leukemic cells, leading to more personalized and effective antileukemia treatment [14,15,16,17,18]. 

This study aimed to investigate the presence, frequency, and predictive value of morphologically identified CLBs at diagnosis in a subsequent series of *NPM1*-mutated patients with AML defined after a fully integrated diagnostic approach, including the real-time identification of *NPM1* mutations and normal karyotypes. The morphological identification of CLB on smears might be a faster and simpler method for the prediction of *NPM1* mutations before all other diagnostic procedures, enabling streamlined genetic work-up for confirmation. This may facilitate considering the early administration of individualized therapies by clinicians for specific patients.

## 2. Materials and Methods

### 2.1. Patients 

This retrospective study is based on a clinicopathological database comprising 397 patients with AML (75 with typical RCA and 322 patients with all AML types) that underwent the full diagnostic workup required to fulfill the WHO 2016 classification proposal, within a 5-year period from August 2018 to August 2023, at the Clinic of Hematology, University Clinical Center of Serbia. As the Public Healthcare System in Serbia is mainly centralized, the entire group of patients represents approximately half of Serbia’s population (3.7 million inhabitants in this referral area). 

From the entire group of patients with AML with normal karyotype, 83/212 (39.15%) patients were identified as having AML with *NPM1* mutations after a fully integrated diagnostic work-up, including morphology, immunophenotyping, cytogenetics, and molecular genetic testing. 

The inclusion criteria were as follows: general informed consent at the time of diagnosis, an initial diagnosis of AML based on bone marrow aspiration before any treatment, normal karyotype, representative diagnostic bone marrow aspiration, peripheral blood film available for thorough evaluation of key morphological features of the leukemia population, and other available clinical and laboratory data, including manual white blood cell differential. Patients previously treated for other malignancies were excluded from this study, as were those with AML evolving from myelodysplasia or other preceding hematological malignancies. This study was approved by the Institutional Review Board of the University Clinical Center of Serbia, according to the Declaration of Helsinki and Good Clinical Practice (protocol number III 41004). 

### 2.2. Cytomorphological Analysis 

Two cytomorphologists (LJ and AB) independently evaluated peripheral blood and bone marrow smears. The blast-cell fraction was assessed using an Olympus^®^ BX53 microscope (Tokyo, Japan). In all, 200 nucleated cells in the peripheral blood (PB) and 500 cells in the bone marrow (BM) aspiration slides were estimated at 1000× magnification [19]. 

The detailed examination of key morphological features of leukemia suggestive of *NPM1*-mutated AML was performed according to WHO 2016 classification [6], including necessary staining using May Grünwald Giemsa (Merck^®^, Darmstadt, Germany), myeloperoxidase (MPO, GrahamKnoll method, Sigma-Aldrich^®^, Saint Louis, MO, USA), and α-naphtol acetate esterase (Leucognost ANAE, cytochemical kit, Merck^®^, Darmstadt, Germany). 

**Figure 1:**
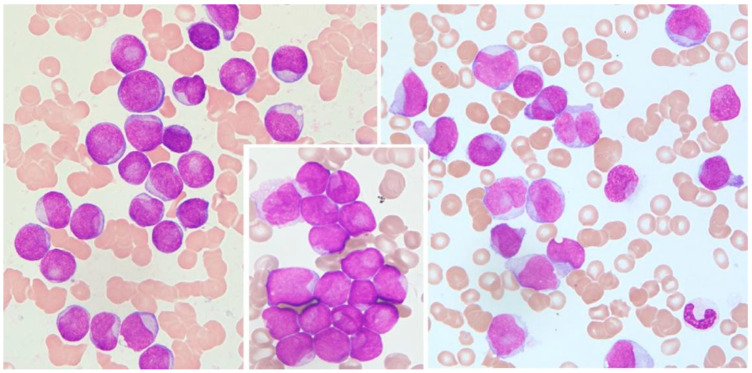
Cup-like blasts in acute leukemia. Left: AML without maturation (MGG, 1000×); middle: detail from AML without maturation (MGG, 1000×); right: AML monoblastic and monocytic type (MGG, 1000×).

Cytological features of interest include prominent nuclear invaginations and CLBs [8,9] due to the indentation of the nucleus by parts of the cytoplasm. The indentation must appear as a pocket or spherical object that compresses an otherwise round or slightly ovoid nucleus, and the compression must be >25% of the diameter of the nucleus (Figure 1). 

This pocket may contain subcellular structures such as mitochondria, lysosomes, and endoplasmic reticulum, which are only visible through electron microscopy [8,20]. All cells with CLB morphology were counted, and the values are expressed as a percent of 1000 counted blast cells. 

### 2.3. Immunophenotyping by Flow Cytometry, Molecular, and Genetic Analysis

Immunophenotyping using flow cytometry (performed on Becton Dickinson (BD) FACS Calibur^®^ (2018–2019) and BD FACS Canto II^®^ (2020–2023) Flow Cytometer, BD Biosciences^®^, San Jose, CA, USA) was carried out systematically according to standard protocols based on ELN Work Package 10 (WP10) criteria [21]. Panels of monoclonal antibodies based on EuroFlow consortium proposals (4–8-colour combinations) were used for the labeling and detection of the immunophenotypic profile of leukemic blast cell populations [22]. Cytogenetic analysis was performed as part of the standard diagnostic procedure, in accordance with the current protocols established for myeloid neoplasms and reported according to the International System for Nomenclature in Human Cytogenetics (ISCN) [23]. 

DNA was extracted from bone marrow samples using a Genematrix quick blood DNA Purification Kit (EURx, Gdansk, Poland). 

The detection of *NPM1* mutations was performed with extracted DNA by the application of Ipsogen *NPM1* MutaScreen^®^ Kit according to procedures proposed by the manufacturer (Qiagen^®^, Hilden, Germany) on a TaqMan ABI Prism^®^ 7500 real-time PCR. This kit detects total *NPM1* (wild-type and mutated) and mutated *NPM1*, and separately identifies *NPM1* Mut A, Mut B, and Mut D in genomic DNA. The Ipsogen *NPM1* MutaScreen Kit provides two results simultaneously: *NPM1* mutation status (mutated or wild-type) and the identification of the *NPM1* mutation type (A, B, or D). 

Testing for *FLT3-ITD* and *FLT3-D835* mutations was performed using PCR-based amplification of genomic DNA, as previously described [24,25]. The PCR products were digested using an EcoRV restriction enzyme. Each PCR product was electrophoresed on 2% agarose gel and visualized under UV light after ethidium bromide staining.

Following the completion of the diagnostic procedures, a joint board of specialists finalized the evaluation. A detailed, independent morphological review of all the patients’ cytological features was performed and evaluated, based on available cytogenetic and other findings, according to the WHO 2016 diagnostic [6] criteria.

### 2.4. Statistical Methods 

To assess the predictive value of morphological evaluation within the established WHO-defined criteria, positive and negative predictive values with confidence intervals (95%) were calculated. Related statistics, sensitivity, specificity, and positive and negative likelihood ratios (LRs) were also examined [26]. Statistical analysis was performed using the 2017 MedCalc^®^ Software bvba (Ostende, Belgium) software package. 

We also applied other statistical methods of descriptive statistics (mean, median, standard deviation (SD)), together with Chi-square and Fisher tests and other nonparametric tests for evaluation of differences between the mean (Mann–Whitney U (MWU) test and Kruskal–Wallis (KW) test). A value of *p* < 0.05 was considered statistically significant. Spearman’s rank order method was used to evaluate correlations. The tests were performed using the statistical software TIBCO Statistica, v13.3 (Palo Alto, CA, USA). Receiver operating characteristic (ROC) analysis was performed on SPSS^®^ software, v17 (SPSS Inc., IBM Corp., Armonk, NY, USA).

## 3. Results 

### 3.1. Correlation between CLB and NPM1 Mutation

Of a total of 397 patients with AML, our study included 212 (53.40%) de novo AML cases with normal karyotype, diagnosed and treated at a single center over a 5-year period. The final diagnosis of *NPM1*-mutated AML, according to the WHO integrated diagnostic criteria, was established in 83/212 (39.15%) patients. Cup-like nuclear morphology, suggestive of *NPM1*-mutated AML, was observed at the time of diagnosis in 56/83 (67.46%) patients with WHO-confirmed *NPM1*-mutated AML. This group comprised 26.41% of all patients with a normal karyotype (56/212). 

Among the 83 patients with AML with *NPM1*, the subtypes were as follows: type A (most common), comprising 66/83 (79.5%); type B, comprising 13/83 (15.6%); and type D, comprising 4/83 (4.9%). 

Discordance between cytomorphological and other integrated criteria in predicting the *NPM1* mutation was detected as a missed/wrong call in 27/2 cases. The accuracy of cytomorphological evaluation was 86.32%.

The positive/negative predictive values of cytomorphological evaluation were 96.55%/82.47%, with a sensitivity/specificity of 67.47%/98.45% for cases with CLB and normal karyotype (N = 212, MedCalc Software). 

**Figure 2:**
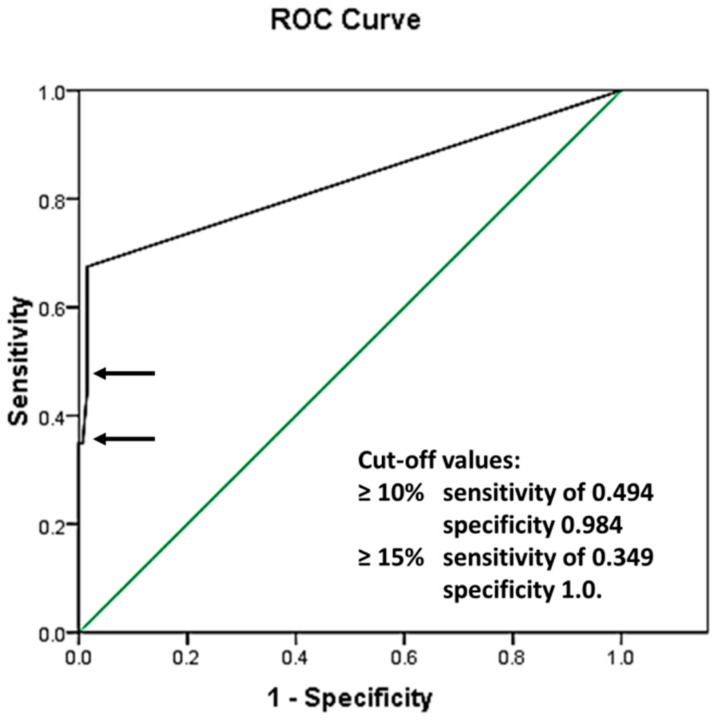
Receiver operating characteristic curve (ROC): area under the curve 0.830 with SE 0.033, and a 95% confidence interval of 0.766 and 0.895. The green line represents a reference line, implying that the analyzed values were separated purely by chance. The black line shows the ROC curve of the analyzed data, representing the relationship between the Sensitivity and 1 − Specificity values of the performed tests. The sensitivity and specificity of the test with cut-off values ≥10% and ≥15% are presented by the top and bottom arrows, respectively.

To evaluate the best CLB cutoff value, ROC analysis was performed in 212 patients with AML with a normal karyotype, demonstrating the following area under the curve: 0.830 with standard error (SE) 0.033, and a 95% confidence interval of 0.766 and 0.895 (Figure 2). 

Cutoff values of 10% were identified as having a sensitivity of 0.494 and specificity of 0.984, and those of 15% were identified as having a sensitivity of 0.349 and specificity of 1.0. Further analysis using the chosen cutoff values is shown in Table 1.

### 3.2. Correlation between CLB and Leukemia Biology

**Figure 3:**
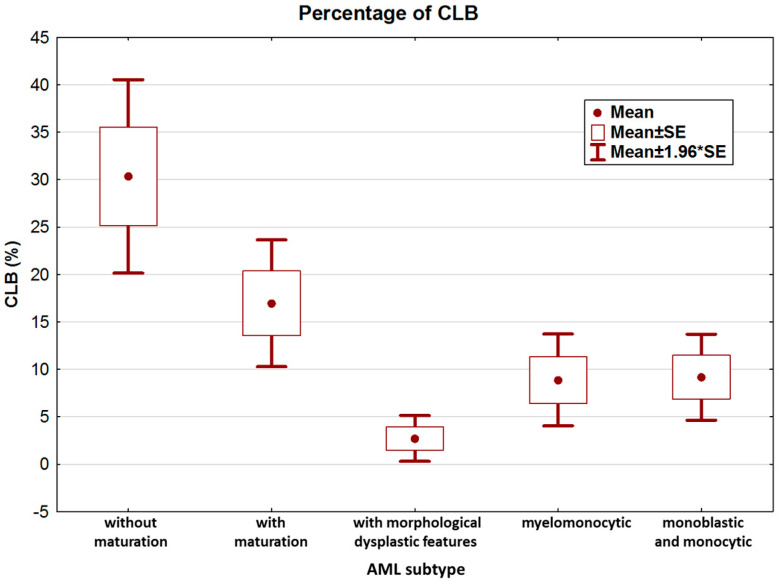
Percentage of CLB according to leukemia subtype.

We further analyzed the clinical and biological features of *NPM1*-mutated AML patients. According to the features and immunophenotype of leukemia, patients were categorized as AML with granulocytic differentiation without maturation (9/83), AML with granulocytic differentiation and maturation (22/83), AML with morphological dysplastic features (20/83), AML with granulocytic and monocytic differentiation (14/83), and AML with monoblastic and monocytic differentiation (18/83). The highest percentage of CLB was observed in the non-mature AML subtype (Figure 3).

Analysis of variance confirmed statistically significant differences in CLB counts among the five groups (KW test, *p* = 0.00001). Paired analysis (MWU test) confirmed that the main differences occurred between AML without maturation and AML with maturation vs. all other groups (Table 2). 

There was no correlation between the *NPM1* subtypes and the types of leukemia analyzed. 

Patients with CLB were most frequent among those with AML without mutation (100%, 9/9), followed by those with AML with mutation (72.7%, 16/22). 

As the ROC analysis confirmed that 10% and 15% cutoff values were highly specific in predicting *NPM1* mutation, we extended our analysis using those values. This revealed that among patients with AML without and with mutation, increased cutoff values resulted in a high percentage of CLB-positive cases compared to other AML subtypes (for ≥10%: Chi-square test, *p* = 0.01; for ≥15%: Chi-square test, *p* = 0.007). 

### 3.3. Correlation between CLB and Different Hematological Characteristics 

Further analysis of all 83 *NPM1*-mutated patients with AML showed that those detected with CLB had a significantly higher WBC count and blast count in PB and BM. We also observed an increase in *FLT3*-ITD and CD117 positivity, whereas HLA-DR expression was significantly lower in CLB-positive cases. Similar results were obtained in the CLB ≥ 10% and CLB ≥ 15% subgroups (Table 3).

Of the 83 patients, 35 were *FLT3*-positive (32 with *FLT3*-ITD and three with *FLT3*-D385). There was a positive correlation between CLB and the presence of *FLT3* mutations, and the Spearman’s rank correlation was significant (*p* < 0.05, R = 0.269). Positive *FLT3* cases had a higher percentage of CLB (16.6 ± 16.3 vs. 9.1 ± 11.8, MWU test *p* = 0.017), but this was not confirmed in stratified analysis as CLB > 10% was detected in 22/35 (63%) patients (Chi-square test, *p* = 0.101; Fisher test, *p* = 0.07). In contrast, CLBs were found in 28/35 (80%) *FLT3*-positive cases (Chi-square test, *p* = 0.03).

In our group, most of *NPM1*-mutated AML patients were CD34-negative (59/83, 71%). Majority of these had CLB morphology as well (40/59, 68%). However, in *NPM1*-mutated AML patients with CD34+ (24/83, 29%), 16/24 were CLB-positive, without statistical significance (Chi square *p* = 0.405). 

No correlation was observed between CLB and CD117 expression (Spearman’s rank correlation, *p* > 0.05), whereas a negative correlation was found between CLB and HLA-DR expression (Spearman rank correlation R = −0.477, *p* < 0.05). Accordingly, most of the analyzed AML cases without and with maturation were HLA-DR-negative with a high CLB count. 

## 4. Discussion

Modern diagnostics for acute leukemia are highly integrative and include classical morphology, immunophenotyping, and genetic data. The approach used for the routine testing of molecular parameters is always a matter of debate, especially over those essential for exact diagnostic classification and treatment [1,2,27]. 

There is no universally standardized genetic test for all recurrent genetic abnormalities and even high-throughput machines have delays [28,29]. Modern cytogenetic/molecular testing can take > 7 days to return results, and there is no consensus regarding the prognostic impact of the time from AML diagnosis to treatment. Advances in genomic profiling, such as next-generation sequencing (NGS), offer the potential to identify therapeutic targets [30], changing the current treatment approaches for patients with AML, particularly for those with a normal karyotype [31]. Currently, NGS panels typically have a slow turnaround time of up to 14 days or more. Moreover, NGS myeloid panels have limited analytical sensitivity towards current real-time PCR testing, especially at low-level burden [32]. This emphasizes the need to create a limited molecular profile, possibly guided by morphological findings, which can reliably predict molecular abnormalities to prioritize or streamline common genomic mutations with a faster turnaround time [28,30].

We previously demonstrated the predictive role of cytomorphological features in the early identification of several common AML-RCAs, particularly AML with t(15;17), before cytogenetic and molecular studies can be completed [33]. 

In the current paper, we apply a similar approach towards *NPM1*-mutated AML cases in a homogenous group of 212 patients with normal karyotype in a single academic center, diagnosed under the same procedures and principles integrated stipulated in the WHO 2016 classification. 

Previous studies demonstrated an association between CLB morphology and *NPM1*-mutated AML [34,35,36]. However, most studies analyzed heterogeneous groups using different inclusion criteria and methodological approaches [8,9,20,37,38,39]. 

Moreover, various CLB cutoff values have been reported, ranging from 5% to 10%, with the latter being more frequent [9,20,28].

Therefore, our investigation performed ROC analysis to determine the sensitivity and specificity of morphological CLB recognition and to determine possible cutoff values suitable for clinical work. Our findings revealed that CLB ≥ 10% is both sensitive and specific in categorizing patients with or without *NPM1* mutation, which is in concordance with some previous publications [9,28,37].

Additionally, our results revealed that the cutoff of CLB ≥ 15% identifies *NPM1* mutations with a high specificity and positive predictive value (100% and 100%, respectively), with the majority of patients belonging to the AML without- and with-mutation subgroups. Although the sensitivity of this cutoff proved to be lower, this could be explained by the fact that most *NPM1*-positive cases with morphological dysplastic features and those with monocytic differentiation presented with lower CLB counts and seldom reached CLB ≥ 10%. 

Further analysis showed an association between the CLB percentage and the presence of *FLT3* in *NPM1*-mutated AML, which is consistent with previous studies [9,28]. We did not notice a statistically significant association between *FLT3* mutation and CLB ≥ 10% and CLB ≥ 15%. Moreover, the negative predictive value of *FLT3* is relatively low, as described previously [28]. 

*NPM1*-mutated AML, although associated with a relatively favorable prognosis when *FLT3* is absent, can still result in treatment failure and relapse in some patients. There is an unmet need to identify optimal frontline therapies for those with *NPM1*-mutated AML. The efficacy of multiple drugs such as venetoclax, menin, and XPO1 inhibitors are under investigation, and several clinical trials have been registered [31,40,41]. As these trials take place, it is essential to consider the use of reliable and easily reproducible tools, such as the morphological identification of CLB, for the early prediction of *NPM1* mutations, streamlining specific, faster, and targeted genetic detection. 

Our study may have limitations arising from the fact that only patients with AML and normal karyotypes were included. However, it has been shown that *NPM1* mutations are seldom observed in patients with high-risk cytogenetic or molecular aberrations [11]. In addition, two wrong call cases were identified during our research. However, it is noteworthy that the real-time PCR kit used in this study detects subtypes A, B, and D of *NPM1* mutations, which account for 88% of cases [42].

## 5. Conclusions

With the advent of novel therapies for *NPM1*-mutated AML, there is a growing need for reliable prediction of *NPM1* mutations. Our investigation confirmed that morphological identification of CLB at diagnosis represents a reliable and easily reproducible tool for the early prediction of *NPM1* mutations, permitting the prioritization of genetic workup for confirmation. This may facilitate the early consideration and administration of individualized therapies by clinicians for specific patients. 

These findings, however, need to be validated in a larger homogenous group of patients with AML. 

## Figures and Tables

**Table 1:** Positive/negative predictive values of cytomorphological evaluation for CLB (n = 212).

CLB	Specificity	Sensitivity	Accuracy	PPV	NPV
≥10%	98.5	53.0	80.7	95.7	76.5
≥15%	100	34.1	73.6	100	0.7

Abbreviations: CLB—cup-like blasts; PPV—positive predictive value; NPV—negative predictive value.

**Table 2:** Distribution and percentage of CLB according to AML subtype.

AML Subtype	CLB (mean/SD)	CLB (Range)	CLB (Median)	Without Maturation vs. Others	With Maturation vs. Others
without maturation	30.3 ± 15.6	12–54	28	n.a.	
with maturation	16.9 ± 16.8	0–50	14.5	*p* = 0.045	n.a.
morphological dysplastic features	4.9 ± 7.1	0–19	0	*p* = 0.0002	*p* = 0.018
myelomonocytic	7.7 ± 10	0–32	1.5	*p* = 0.0018	*p* = 0.139
monoblastic and monocytic	9.2 ± 9.8	0–33	10	*p* = 0.0009	*p* = 0.343

Abbreviations: CLB—cup-like blasts; AML—acute myeloid leukemia; n.a.—non applicable.

**Table 3:** Selected characteristics of *NPM1*-mutated AML according to CLB findings.

Features	CLB Positive	*p* Value	CLB ≥ 10%	*p* Value	CLB ≥ 15%	*p* Value
WBC (mean/SD)	69.5 ± 76.6 vs. 30.6 ± 43.8	*p* = 0.009	83.7 ± 80.5 vs. 26.6 ± 33.7	*p* = 0.00052	76.9 ± 67.0 vs. 67.1 ± 69.6	*p* = 0.014
FLT3+ vs. FLT3− (%)	16.6 ± 16.3 vs. 9.06 ± 11.8	*p* = 0.017	63 vs. 46	*p* = 0.094	46 vs. 27	*p* = 0.07
HLA-DR+ (%) (mean/SD)	57.7 ± 17.3 vs. 82.9 ± 40.7	*p* = 0.013	52.7 ± 40.8 vs. 80.9 ± 24.5	*p* = 0.0009	39.6.7 ± 38.2 vs. 80.1 ± 27.1	*p* = 0.00001
PB blasts (%) (mean/SD)	51.7 ± 35.9 vs. 17.1 ± 16.8	*p* = 0.00005	60.8 ± 34.5 vs. 17.5 ± 16.5	*p* = 0.00001	70.3 ± 28.8 vs. 24.4 ± 26.5	*p* = 0.00001
BM blasts (%) (mean/SD)	67.9 ± 20.4 vs. 48.5 ± 18.6	*p* = 0.0002	71.5 ± −20.2 vs. 50.6 ± 17.9	*p* = 0.00007	74.6 ± −9.2 vs. 54.4 ± 19.7	*p* = 0.00004

Abbreviations: CLB—cup-like blasts; AML—acute myeloid leukemia; WBC—white blood cell; PB —peripheral blood; BM—bone marrow.

## Data Availability

The data that support the findings of this study are available on request from the corresponding author.
